# The peculiar characteristics and advancement in diagnostic methodologies of influenza A virus

**DOI:** 10.3389/fmicb.2024.1435384

**Published:** 2025-01-07

**Authors:** Muhammad Asif Raza, Muhammad Awais Ashraf, Muhammad Nabeel Amjad, Ghayyas Ud Din, Bei Shen, Yihong Hu

**Affiliations:** ^1^CAS Key Laboratory of Molecular Virology and Immunology, Institutional Center for Shared Technologies and Facilities, Pathogen Discovery and Big Data Platform, Shanghai Institute of Immunity and Infection, Chinese Academy of Sciences, Shanghai, China; ^2^University of Chinese Academy of Sciences, Beijing, China

**Keywords:** influenza A virus, diagnosis, polymerase chain reaction, next generation sequencing, nucleic acid sequence-based amplification, loop-mediated isothermal amplification

## Abstract

Influenza A virus (IAV) is a significant public health concern, causing seasonal outbreaks and occasional pandemics. These outbreaks result from changes in the virus’s surface proteins which include hemagglutinin and neuraminidase. Influenza A virus has a vast reservoir, including wild birds, pigs, horses, domestic and marine animals. It has over 130 subtypes based on differences in hemagglutinin and neuraminidase protein. IAV affects all age groups but impacts young children more especially during the colder season. Despite the development of vaccines and antiviral drugs, IAV is still a major cause of respiratory illnesses and deaths. Surveillance of IAV is crucial to detect new strains and assess vaccine effectiveness. Detection of IAV relies on methods like hemagglutination assay, PCR, cell culturing, and immunochromatography-based tests. Precise and early detection of IAV strain is crucial for quick treatment using antiviral drugs and unraveling epidemiological patterns to curb epidemics and pandemics on time. Advancements in diagnostic methodologies have enabled us to detect the IAV at early stages by overcoming the limitations of previously used diagnostic tests, further preparing us to combat future epidemics more effectively. This review article discusses the traditional and advanced diagnosis methods for detecting IAV.

## 1 Introduction

Influenza, commonly known as flu, is a respiratory disease caused by viruses belonging to the family *Orthomyxoviridae*. This family is subdivided into four genera (*Influenza virus A, Influenza virus B, Influenza virus C, and Influenza virus D*). They are single-stranded negative-sense RNA viruses (Böhm et al., 2021). IAV can infect humans, pigs, birds, and other animals. It is responsible for seasonal epidemics which cause 3 to 5 million severe cases and approximately 290,000 to 650,000 respiratory deaths annually worldwide. IAV is responsible for sporadic pandemics globally because of antigenic shifts and antigenic drifts of their neuraminidase (NA) and hemagglutinin (HA) surface proteins (Macias et al., 2021). Novel strains of IAV emerge when genes are reassorted between avian, human, or swine influenza species, and these novel IAVs are transmitted to humans from the reservoir species (i.e., swine, birds). In the past, IAV pandemics emerged in 1918, 1957, 1968, 1977, and 2009, of which the worst was the 1918 Spanish flu which killed more than 50 million people globally (Macias et al., 2021). IAVs are categorized into subtypes based on HA and NA surface proteins. Up till now, 18 HA and 11 NA have been identified, while more than 130 IAV subtypes have been identified primarily residing in wild birds. A highly pathogenic avian influenza virus (HPAIV) H5N1 has been circulating since 1997. It was first isolated from Hong Kong with a mortality rate above 60% (Reperant and Osterhaus, 2022).

IAVs are of particular concern due to their ability to cause pandemics, unlike influenza B, C, and D viruses, which primarily cause seasonal outbreaks and have limited pandemic potential. The frequent reassortment of IAV, particularly in regions where humans and animals live in close proximity, increases the likelihood of novel viral strain emergence. This makes IAV a critical focus in public health strategies, emphasizing the need for continuous ongoing surveillance, vaccine development, and preparedness efforts to mitigate the impact of future pandemics (Reperant and Osterhaus, 2022). Diagnosis of IAV in clinical settings is very important to early detect the onset of disease and combat the infection effectively. Surveillance of IAV is also essential to detect any novel and emerging IAV strain in human as well as animal populations. In this review article, we have discussed the methods that have been developed for the identification of IAV. We have briefly covered the characteristics of IAV and comprehensively covered the classic as well as advanced methodologies for the diagnosis of the influenza A virus.

## 2 Structural composition

**Figure 1:**
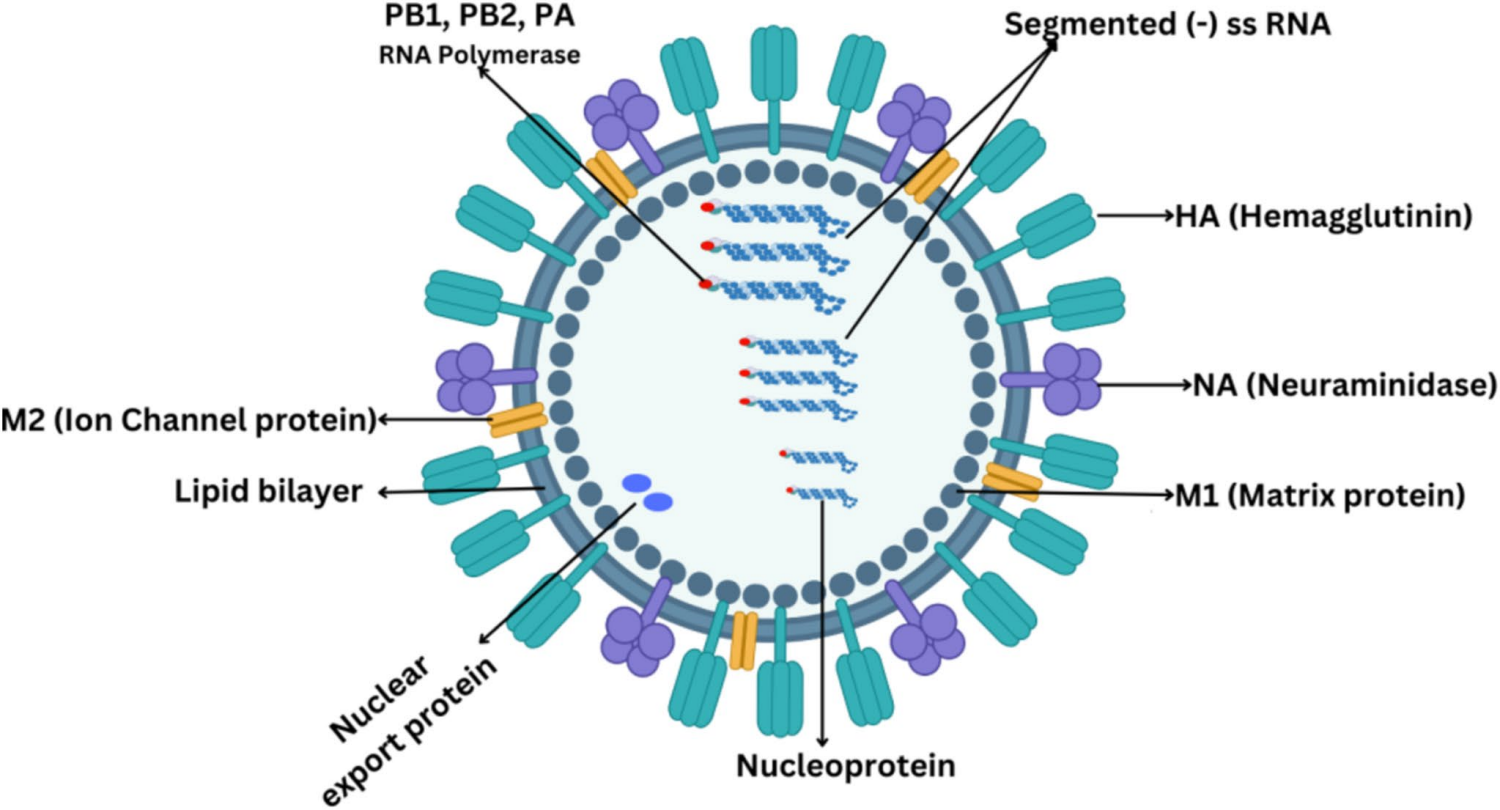
Structural composition of IAV.

IAVs are morphologically highly diverse, with spherical diameters ranging from 80 to 100 μm to filamentous with a length of several micrometers (Knipe and Howley, 2013). The overall virion structure comprises a host-derived membrane containing viral integral proteins, namely hemagglutinin, neuraminidase, and matrix M2 protein as shown in Figure 1. The HA protein appears as a trimer and exhibits a spike on the virus’s surface. The NA protein leaves the membrane as a tetramer and appears as a globular shape. The matrix M1 protein is primarily associated with determining the virion shape (Bourmakina and García-Sastre, 2003). M1 protein represents a bridge between viral RNA and the polymerase complex and between nucleoprotein and lipid membrane. The M2 protein is a short protein with a critical role as an ion channel protein that initiates viral uncoating (Moriyama et al., 2019).

All IAVs comprise eight gene segments, each segment coding at least one protein. The proteins that form the structure of IAV can be divided into two categories. One is the surface proteins, which include hemagglutinin (HA), neuraminidase (NA), and ion channel proteins (M2). Second is the internal proteins which include nucleoproteins and matrix proteins (M1). Three kinds of nucleoproteins are polymerase basic protein 1 (PB1), polymerase basic protein 2 (PB2), and polymerase acidic protein (PA). M1 is the most abundant protein in the influenza A virus which forms the endoskeleton of viron just under the lipid bilayer as shown in Figure 1 and mediates virus assembly (Peukes et al., 2020). IAV produces several non-structural proteins (NSPs), including nuclear export proteins (NEPs), nonstructural protein 1 (NS1), and nonstructural protein 2 (NS2), which are crucial in the virus’s life cycle. NS1, a true nonstructural protein, is abundantly produced within infected host cells but is not encapsulated within the viral particle (Tumpey et al., 2005). Recent discoveries have expanded the list of NSPs to include PA-X, PB1 frame 2 (PB1-F2), PB1-N40, PA-N155, PA-N182, M42, and NS3. These NSPs primarily arise from splicing, frameshifting, and truncation events within the coding regions of structural proteins. While most of these NSPs, except NS2, are non-essential, they play significant roles in suppressing host defenses, contributing to virulence, and enhancing pathogenicity. NS1 is particularly well-characterized for its multifaceted role in inhibiting host innate immunity, shutting off host gene expression, and facilitating viral replication. It exerts its role in activating the PI3K/Akt pathway, which delays virus-induced apoptosis, thereby allowing sufficient time for viral replication. Additionally, NS1 acts as a key virulence determinant in the IAV life cycle (Akarsu et al., 2003; Goraya et al., 2020). The NS2 protein, encoded by segment 8 of the IAV genome, is derived from a spliced mRNA transcript and is highly conserved across all sequenced IAV strains. Unlike other NSPs, NS2 is essential for the IAV life cycle. NS2 plays a critical role in vRNP nuclear export and is pivotal for viral RNA transcription and replication. Its functional significance and presence in both the host cell and viral particle distinguish NS2 from other NSPs (Hao et al., 2020).

## 3 Epidemiology

### 3.1 History

**Figure 2:**
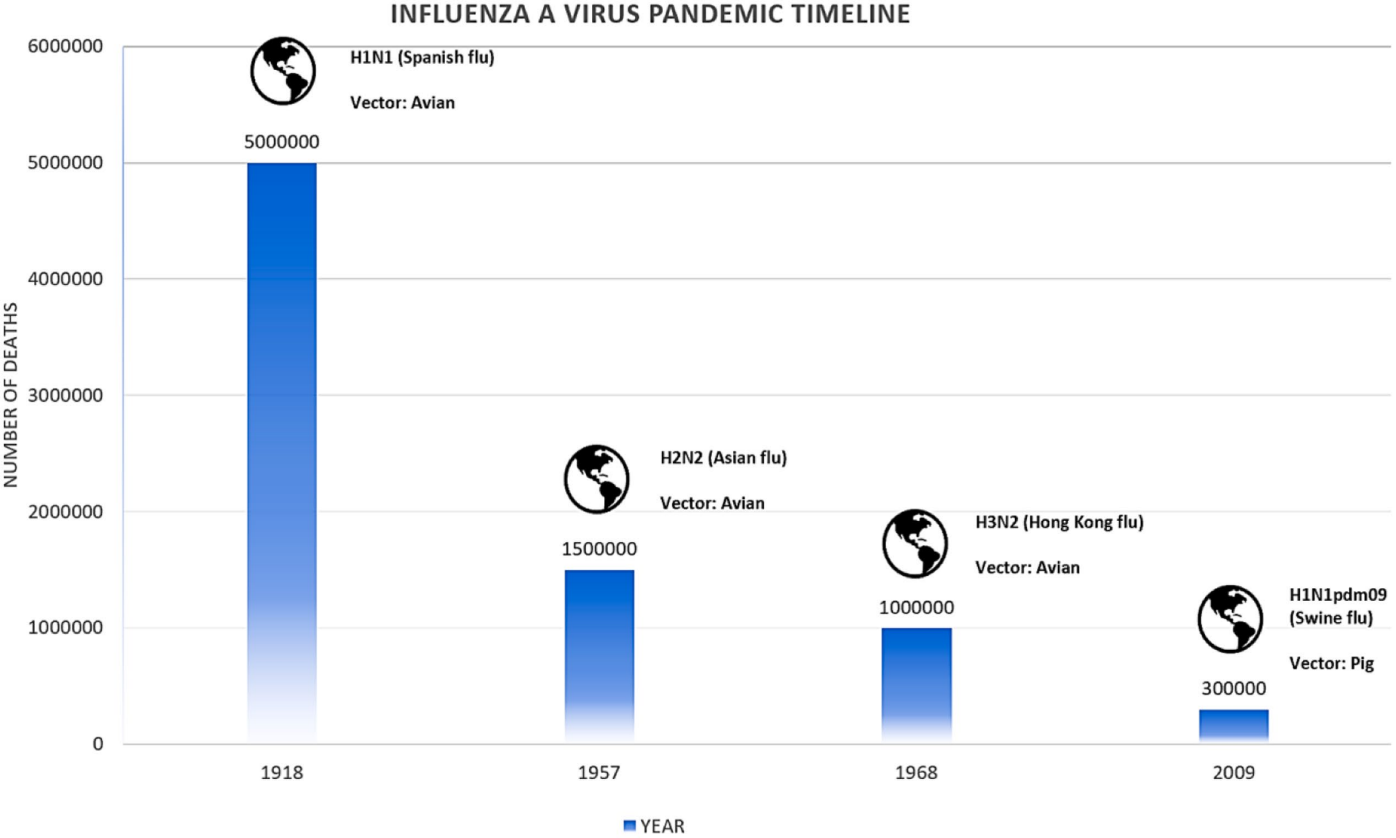
Major pandemics caused by IAV.

The 1918 influenza pandemic, often referred to as the “Spanish Flu,” stands out for its unusually high mortality rate among young adults aged 20–39 years. This unexpected pattern is largely attributed to an abnormal immune response, with some evidence suggesting that older individuals might have been partially protected due to prior exposure to a similar virus. Contrary to the typical W-shaped epidemic curve, which includes high mortality rates in both the very young and the elderly, the 1918 pandemic was distinct in its impact on younger adults (Shepherd, 2023). In 1957, a new influenza A subtype H2N2 emerged through genetic reassortment, combining segments from an avian virus with the existing H1N1 virus, known as “Asian influenza.” This pandemic replaced the IAV H1N1 and primarily affected children and the elderly (Monto and Fukuda, 2020). In 1968, another reassortment event led to the emergence of the IAV H3N2, which replaced the H2N2 strain. This pandemic is often referred to as the “Hong Kong flu” as shown in Figure 2. The frequent occurrence of reassortment events in East Asia, where humans, poultry, pigs, and wild birds live in close proximity, led to the belief that this region is a breeding ground for new influenza viruses. In 2009, a novel IAV H1N1, distinct from previous strains, emerged in Mexico, marking the first influenza pandemic of the 21st century. The 2009 pandemic disproportionately affected younger individuals, with older populations likely protected by prior exposure to earlier H1N1 strains (Jhung et al., 2011).

### 3.2 Seasonality and transmission

**Figure 3:**
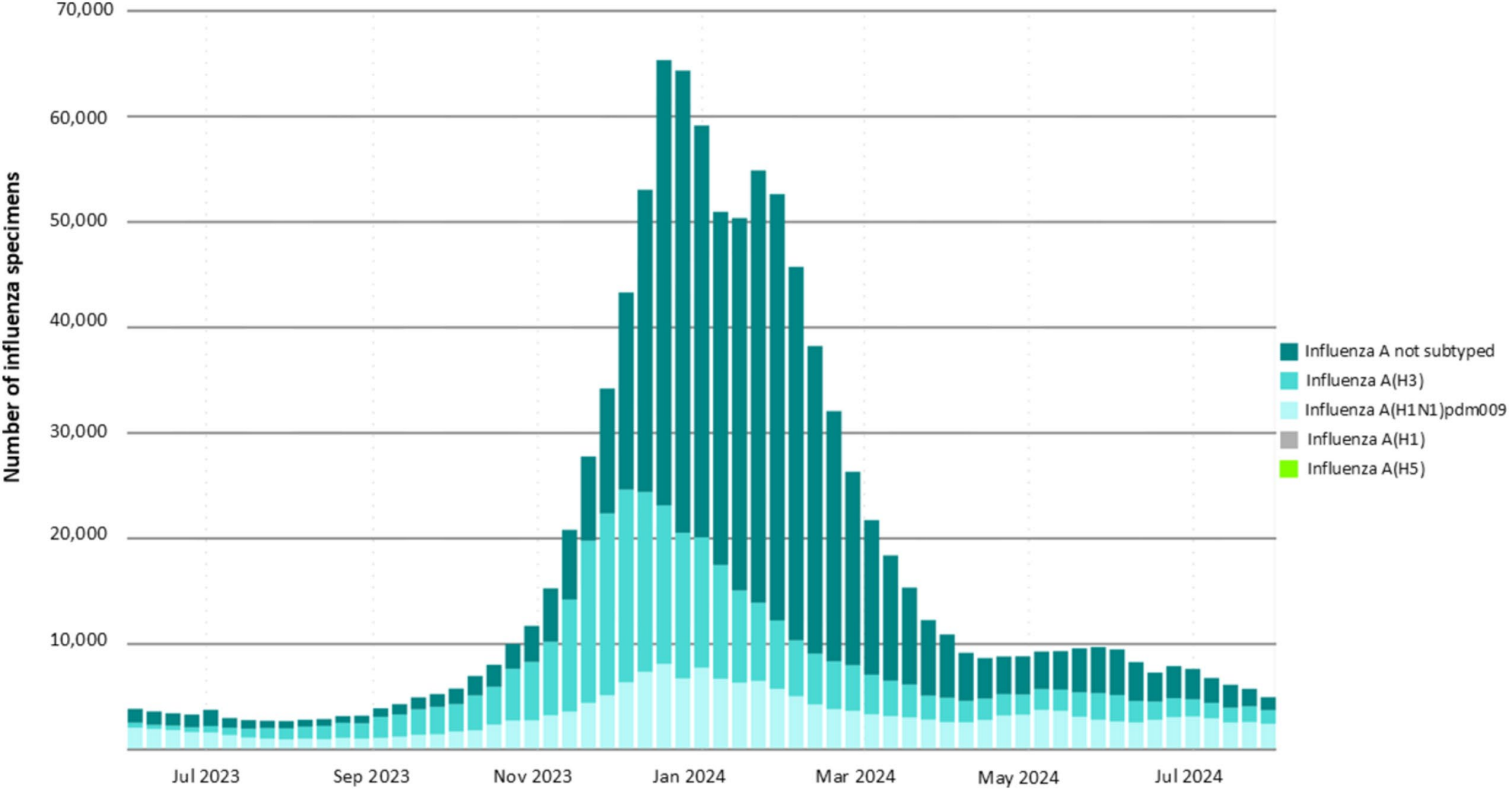
Global surveillance of IAV from July 2023 to July 2024.

In temperate regions of the northern and southern hemispheres, influenza virus infection cases tend to peak during the winter. In contrast, in tropical zones, infection rates surge during the wet season. This increase in infections is attributed to factors such as cool, wet weather, overcrowding in enclosed spaces, longer survival of the virus in aerosolized air, and moist conditions that facilitate virus transmission (Durand et al., 2015). Recent global data on influenza A virus surveillance from June 2023 to July 2024 taken from the fluNet shows that influenza cases peaked during the winter season. In this period, the H3 strain was more prevalent around the globe followed by the H1 strain as shown in Figure 3. A recent surge in HPAIV H5N1 has been seen in wild birds, having pandemic potential. A study in the USA has found H5N1 in dairy cows, their unprocessed milk, and human infections, highlighting the virus’s ability for inter-species transmission (Uyeki et al., 2024).

IAV spreads through droplets expelled from patients during coughing, sneezing, or exhalation, potentially infecting susceptible individuals. The sudden explosive surge in influenza A virus infections is due to the shedding of the virus in nasopharyngeal secretions via aerosols, high titer viral load, and a shallow incubation period of about 2 to 3 days. A typical pattern has been observed during influenza outbreaks: the target susceptibles are young school-going children, compared to adults who already have cross-reacting antibodies and herd immunity (Tian et al., 2015). Findings show that avian influenza viruses spread from one continent to another continent by migratory birds. A bird’s migratory pattern shows better-sequenced data of viral genome sequence than other means (i.e., bird trade), which contributes to the seasonal influenza pandemic at a local level. Moreover, Siberia is the main hub for the transmission of viruses through migratory birds. Birds’ spread of the influenza A virus has increased the risk of exposure to novel strains in humans (Mathur et al., 2014).

## 4 Host range

**Figure 4:**
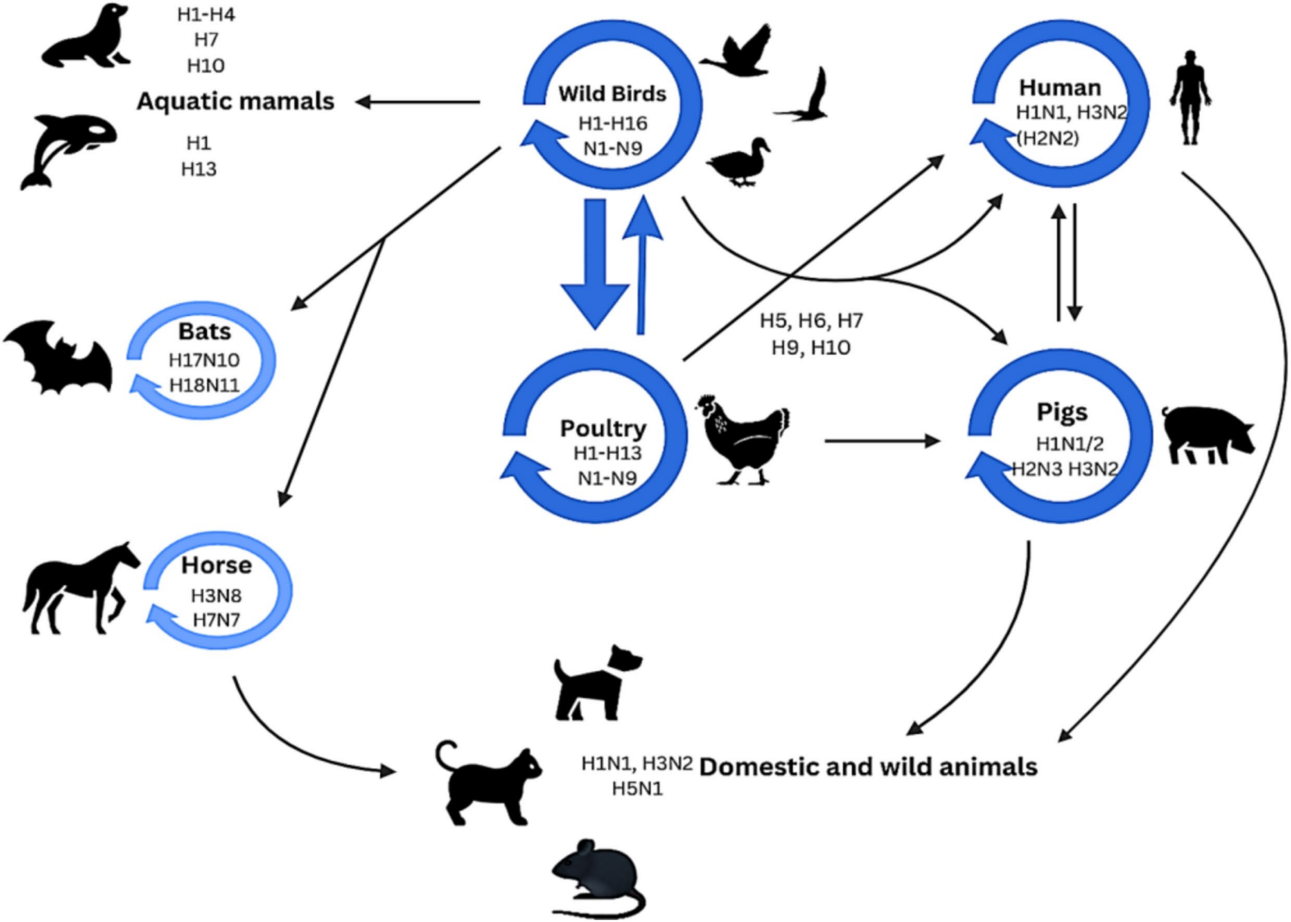
Host reservoir and inter-species transmission of IAV strains.

A vast and antigenically diversified reservoir of influenza A virus is found in wild aquatic birds that normally show no symptoms. Avian influenza viruses can also infect different species. Avian influenza viruses have been seen to spread from birds to various species, including humans. Subtypes from their reservoir can inter-cross with other species as indicated by the dark blue circles in Figure 4. This process can also be accomplished via an intermediate host, and a mutation may be needed for this cross. Certain subtypes of influenza dominate in specific species, as indicated by light blue circles in Figure 4 (Long et al., 2019).

## 5 Diagnostic methods for influenza A virus

World Health Organization (WHO) suggests that reverse transcriptase polymerase chain reaction (RT-PCR) should be used for the diagnosis of influenza A virus because this PCR technique will differentiate IAVs from other influenza virus types. Several methods, including hemagglutination assay, ELISA, egg and tissue culture inoculation, neuraminidase inhibition tests, agar gel immunodiffusion assay (AGID), and RT-PCR, are advised by the WHO for the detection of IAVs (Alexander, 2008).The choice of diagnostic methods depends on the laboratory facilities, training of the lab personnel, resources, and required time. All the traditional and advanced diagnostic methods have benefits and drawbacks, which one must consider when performing a test.

### 5.1 Traditional methods

#### 5.1.1 Virus culture

**Figure 5:**
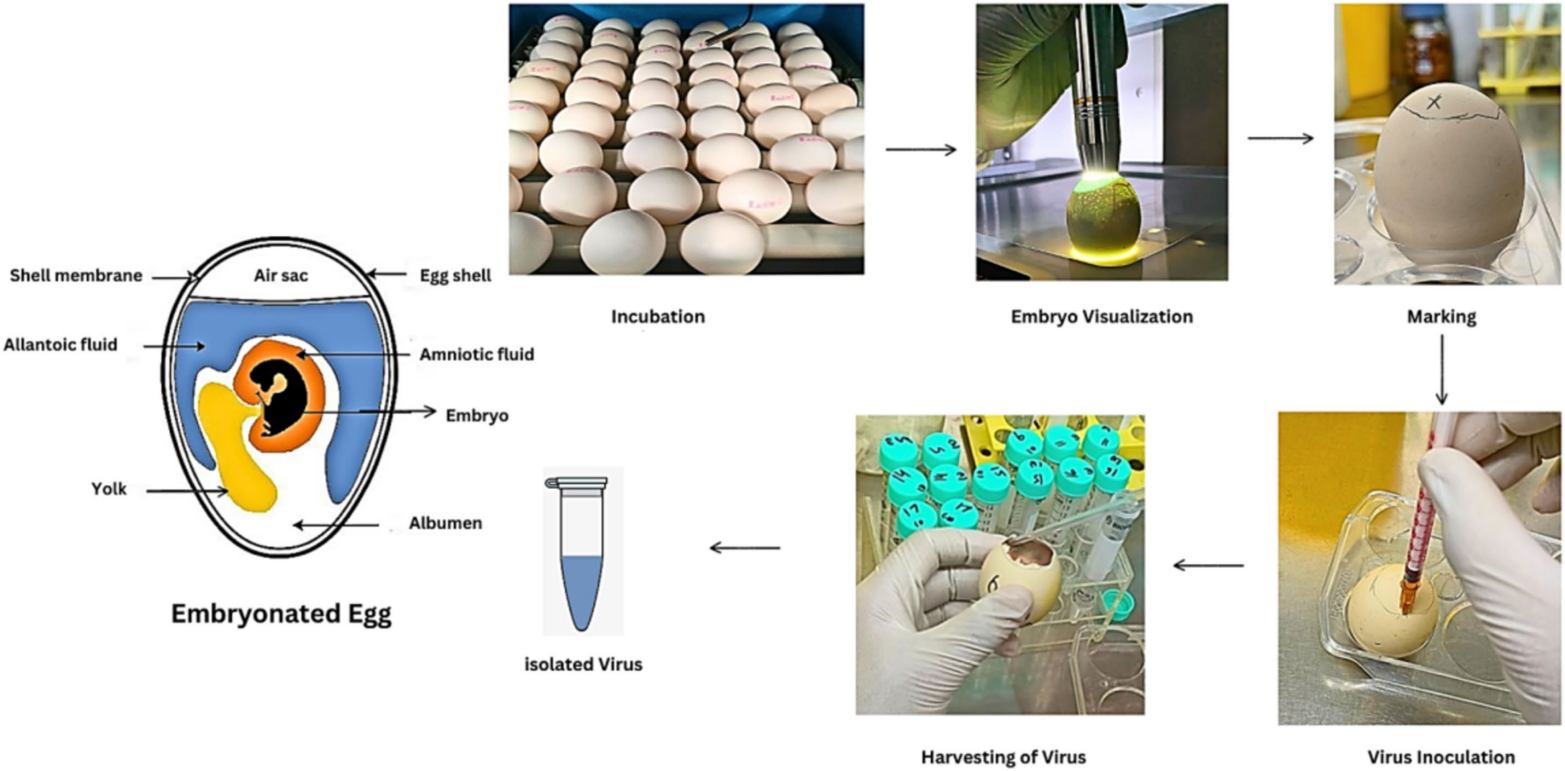
Influenza A virus culture in embryonated chicken eggs.

Influenza A virus is grown using cell lines or embryonated chicken eggs, depending on the source of sample collection. Before the availability of cell culture, human and swine IAVs were grown in embryonated chicken eggs. Still, avian IAVs are typically grown in embryonated chicken eggs due to their suitability for IAV growth (Figure 5). Avian IAVs contain HA protein that binds with alpha 2,3 linked sialic acid moieties and are abundant in cells lining the allantoic cavity of embryonated chicken eggs. This makes the allantoic cavity an ideal medium for their growth (Na et al., 2024). On the other hand, human influenza A viruses prefer to bind with alpha 2,6 sialic acid. This is why they proliferate in the amniotic cavity of embryonated chicken eggs, where alpha 2,6 and 2,3 sialic acids are present (Carter and Iqbal, 2024).

Alternatively, IAVs are grown in the cell lines. At present, multiple cell lines are available for the growth of viruses. The Madin-Darby Canine Kidney (MDCK) cell line is preferred for culturing IAVs. Adding trypsin in MDCK culture media increases the propagation abilities of IAV compared to the MDCK culture media without trypsin. Trypsin facilitates the cleavage of the precursor viral surface glycoprotein HA0 into the HA1 and HA2 subunits, which enhances viral proliferation in MDCK cells. This event is essential for the HA to fuse with the endosome’s membrane and for the genetic material to be released into infected cells (Skehel and Wiley, 2000). MDCK cells display alpha 2,6 and alpha 2,3 sialic acid linkages on their surfaces, making them an ideal growth medium for IAVs that do not proliferate well in chicken embryos. To enhance the titer of IAVs in culture, MDCK cells have been engineered to possess more 2,6-linked sialic acid receptors on their surfaces. This facilitates the culturing of IAV more efficiently (Hatakeyama et al., 2005).

The development of suspension cell culture technology in recent years has streamlined the culturing process of IAV by eliminating the need for trypsinization and cell reattachment, which are typically required in microcarrier-based and Petri plate systems (Peschel et al., 2013). In suspension cultures using serum-free MDCK-SFM2, cells demonstrated faster proliferation, achieved a higher peak viable cell density, and showed reduced cell death, indicating improved cell growth conditions. The results of the study showed that the HA and TCID50 titers of cultured IAVs were significantly higher in the MDCK-SFM2 suspension culture compared to other tested conditions, signifying more efficient influenza virus production. The growth performance of MDCK cells in the serum-free medium MDCK-SFM2 was found to be comparable to that observed in adherent serum-containing cultures as well as in suspension cultures using commercially available serum-free media. Importantly, influenza H1N1 virus production was significantly enhanced in the MDCK-SFM2 suspension culture system (Huang et al., 2015).

#### 5.1.2 Hemagglutination assay

**Figure 6:**
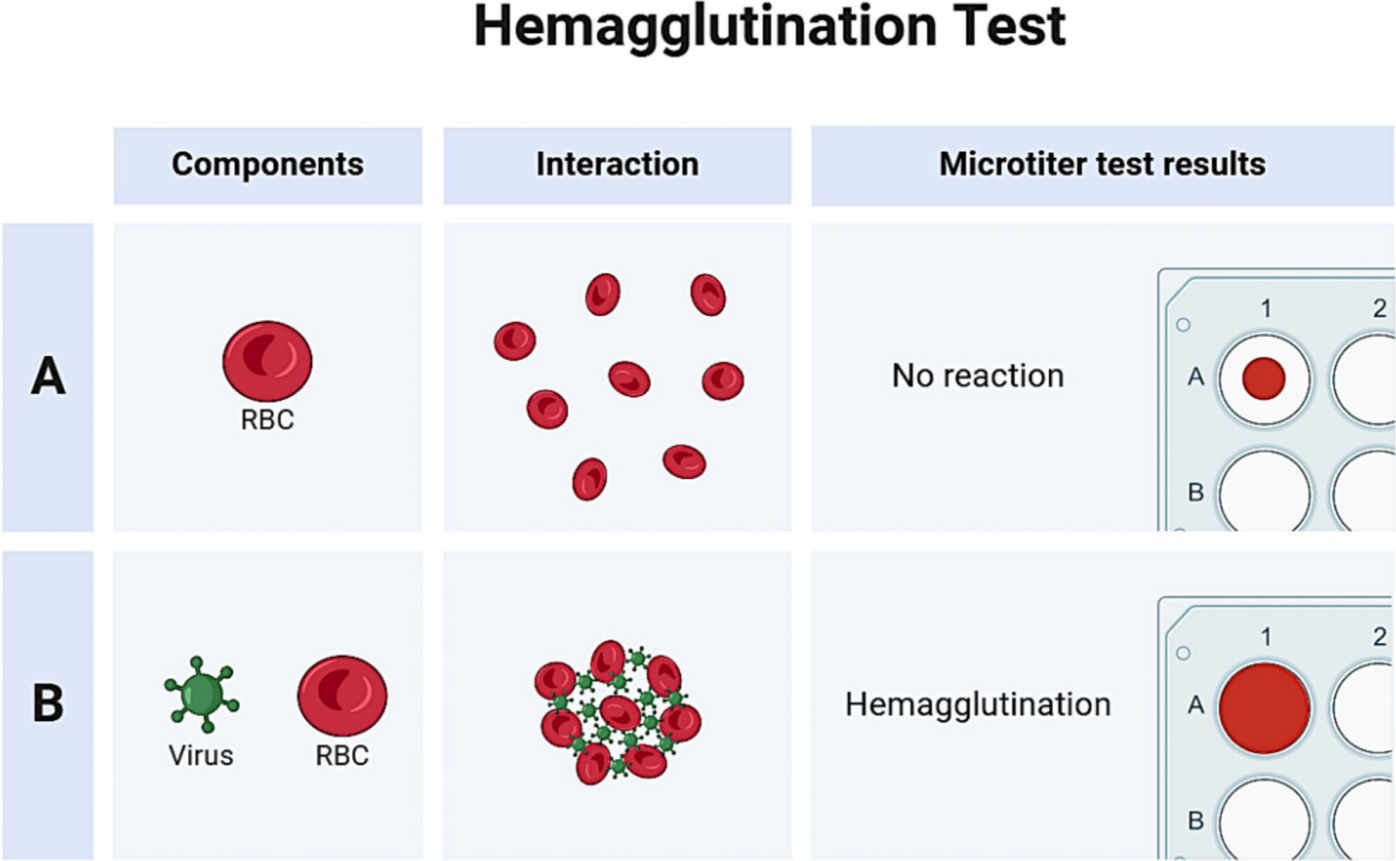
HA test for the presence of IAV in the sample.

The hemagglutination assay, first devised by Hirst and Francis et al., is a commonly used, affordable, quick, and standard screening method for diagnosing and titering influenza A virus. The basic working principle of the hemagglutination test is the ability of the viral hemagglutinin protein to adhere to and agglutinate red blood cells (RBCs), as shown in Figure 6. Hemagglutinin is bound to RBCs by the 2,6 and/or 2,3-linked sialic acid moieties on their surface. In hemagglutination assay, virus samples are diluted with a standard quantity of RBCs in micro-titer plates. Agglutination is observed after an incubation period of around 30 min. Normally, RBCs form a “button” or “halo” at the bottom of a well, surrounded by a transparent buffer. However, when RBCs clump together during agglutination, they appear hazy (Townsend et al., 2021).

Hemagglutination tests are often weakly positive for IAV and HA protein in samples collected during surveillance; however, this assay helps screen various viral cultures before doing further, more specialized diagnostic investigations (e.g., RT-PCR; Sawant et al., 2023). Avian RBCs (such as turkey) are a standard choice in hemagglutination experiments because they are heavy, have a nucleus, and settle quickly in micro-titer plates. As a result, negative activity may be easily distinguished from positive agglutination activity. However, anucleated mammalian RBCs (such as those from guinea pigs and humans), which are lighter, can make halos when settling, potentially confusing the distinction between negative and positive activity. The source of RBCs used in hemagglutination assays should be carefully selected based on the anticipated presence of the viruses in light of these factors (Pawar et al., 2012). A sample known to contain an IAV with active agglutination activity (positive control) and virus-free cell culture medium or egg fluids (negative control) are both helpful as a control for hemagglutination experiments. Although the hemagglutination assay has certain limitations, it is still used in many laboratory settings for diagnostic purposes. The main drawbacks of this assay include its non-specificity, as red blood cells can also agglutinate with other enveloped viruses, such as coronaviruses and paramyxoviruses (Debski-Antoniak et al., 2024). Additionally, the assay lacks sensitivity to detect low viral loads in samples and does not provide quantitative data on viral load.

#### 5.1.3 Immunochromatography

**Figure 7:**
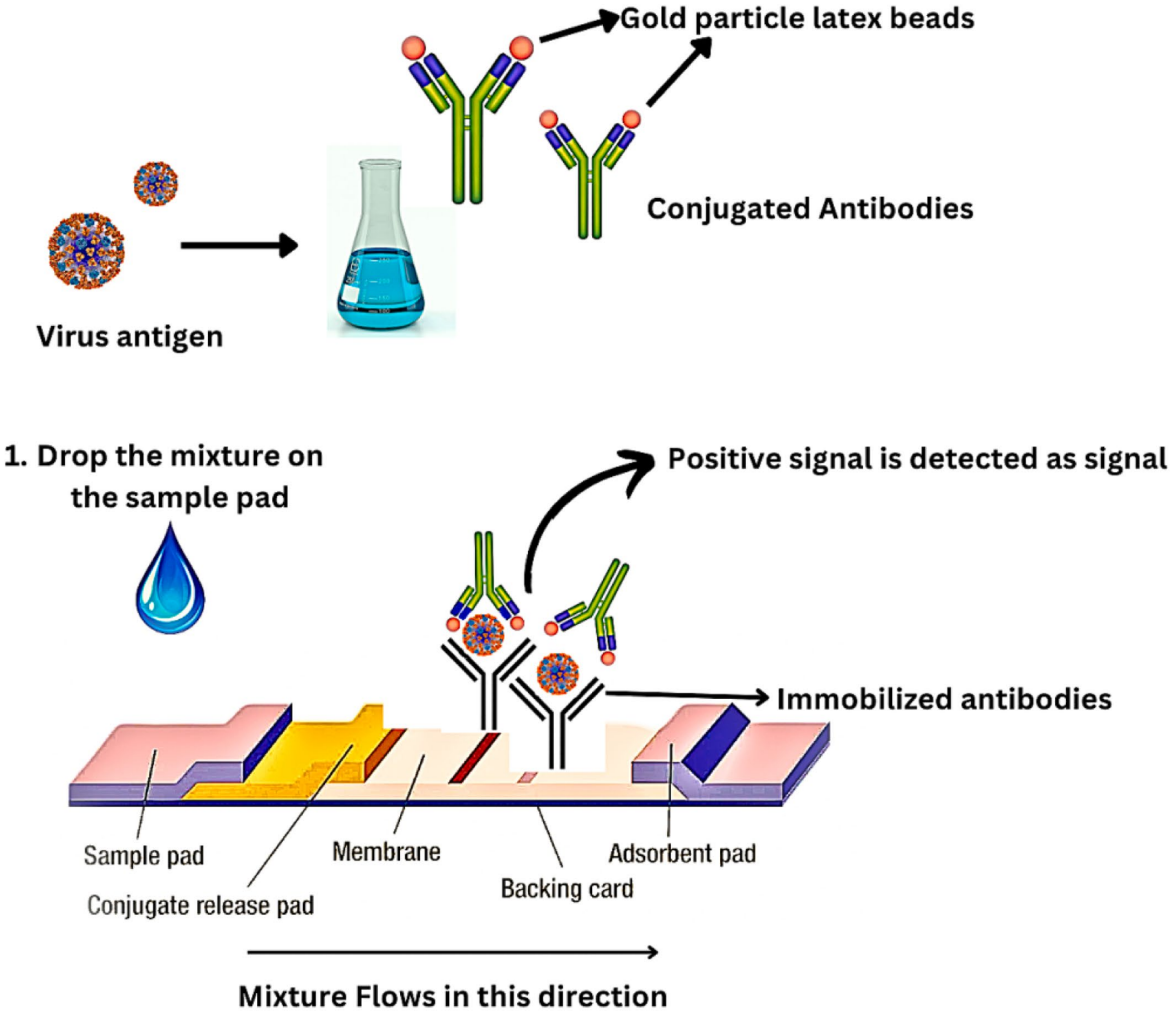
Mechanism of immunochromatography.

Immunochromatography (IC) is a rapid antigen-based technique that takes around 30 min to perform. It is a rapid test for clinical diagnosis and surveillance of IAVs (Sakai-Tagawa et al., 2010). ELISA and IC operate on the same fundamental principles. Nasal swabs from the patient are obtained and immediately mixed with a particular viral antigen–antibody in addition to colloidal gold which is a colored latex-like enzyme. An immobilized viral antigen–antibody is applied to a sample pad on a nitrocellulose membrane as part of the antigen capture procedure, expressing a positive signal (Figure 7).

Although IC is quick and simple, it has a relatively low sensitivity. The specificity of IC is more than 90%, while the sensitivity is just 60%. As a result, this approach is ineffective for detecting IAVs in the early stage of infection. IC sensitivity can be enhanced by conjugating the antibody with fluorescent beads, quantum dots, or other fluorescent components (Khreich et al., 2010). The IC strip is read by a fluorescence reader and scanned. Findings have shown that the fluorescent IC method has a sensitivity that is 50 times higher than traditional IC methods. Additionally, it is possible to quantify the fluorescence IC. For fluorescent imaging, specialized scanners such as an image analyzer and fluorescence reader are required. However, because of their quick detection and high specificity, improved IC techniques are anticipated to be crucial in the surveillance and diagnosis of IAVs (Sakurai et al., 2015).

### 5.2 Advanced methods

#### 5.2.1 PCR-based methods

##### 5.2.1.1 Reverse transcriptase polymerase chain reaction

Reverse transcriptase polymerase chain reaction (RT-PCR) is a standard PCR method that provides a rapid and reliable diagnosis and typing of influenza A virus. To diagnose IAV, viral RNA is first extracted from the sample, and then complementary DNA (cDNA) is synthesized using an enzyme called reverse transcriptase. Specific primers are then used to amplify this cDNA. The first 12 nucleotides of the 3′ terminus (Uni12) and the first 13 nucleotides of the 5′ terminus (Uni13) are conservative in all eight segments of the IAV gene. Thus, all IAV segments amplify the Uni12 (5’-AgCAAAAg CAgg-3′) and Uni13 (5’-TCATCTTTgTTCC-3′) sequences in a single step (Bi et al., 2016). Using RT-PCR, primers specifically Uni12 and Uni13, effectively identify IAV in human nasal swabs. The subsequent sequencing analysis of these samples provides reliable information about the viral subtypes (Sakurai and Shibasaki, 2012). Due to the variability in the sequences of the HA and NA segments, subtype-specific primers are required for the RT-PCR amplification process used in IAV subtyping. Additionally, degenerate RT-PCR has been employed for subtyping IAV NA genes (Qiu et al., 2009). Although RT-PCR is sensitive and provides quantitative results, it is expensive and requires highly skilled personnel to perform.

##### 5.2.1.2 Quantitative real-time polymerase chain reaction

Quantitative real-time polymerase chain reaction (rt-qPCR) is commonly used for viral detection and subtyping of IAVs. This technique uses fluorescent molecular or chemical dyes coupled with molecular oligonucleotide probes to stain the PCR products. The TaqMan probe is one of the examples. Its 5′ and 3′ termini are covalently coupled with a fluorophore and a quencher, respectively. In the PCR products, this causes fluorescence quenching and annealing with the particular region. Taq polymerase extends PCR primers, and its 5′ to 3′ exonuclease activity breaks down the probe that has been annealed to the PCR template. Fluorescence is observed in a dose-dependent manner in the PCR product when the fluorophore and quencher separate due to probe degradation, indicating the presence of IAV in the sample (Ryazantsev et al., 2012). The SYBR green technique is a typical rt-qPCR procedure that interacts strongly with double-stranded DNA and RNA but weakly with single-stranded DNA and RNA. Due to its high sensitivity, specificity, and quantification range, rt-qPCR is useful for diagnosing and monitoring IAVs. It emits green fluorescence through the DNA/RNA-SYBR green complex in the presence of IAV in the sample, with the fluorescence intensity increasing as the amount of PCR product increases. While detecting IAV, PCR amplification targets the matrix gene region of the virus, which is conserved in various subtypes (Habib-Bein et al., 2003). The TaqMan method’s sensitivity makes it possible to identify IAV infections in humans and birds. In contrast, the SYBR green technique demands that the reaction conditions be adjusted due to the lack of a specific probe. Additionally, IAV subtypes and small sequence variants of several matrix genes can be distinguished using melting curve analysis using the SYBR green method (Krafft et al., 2005).

##### 5.2.1.3 Super high-speed quantitative real-time polymerase chain reaction

An innovative kind of real-time qPCR called super high-speed quantitative real-time polymerase chain reaction (SHRT-PCR) has a short turnaround time of less than 20 min for 40 cycles. The reaction mixture is transferred and rotated using a thin, compact disk-style sample container at three distinct temperatures. As it distinguishes between viral RNA segments of IAV, particularly the H5N1 subtype, this approach is useful for diagnosing and detecting IAV infections. However, it can only hold 12 samples. In the future, a “new generation” real-time qPCR method is anticipated to build on the faster response rate of SHRT-PCR (Sakurai et al., 2011).

##### 5.2.1.4 Multiplex PCR

Multiplex PCR is a type of real-time PCR that involves multiple DNA/RNA amplifications using different primer sets in a single tube. Multiplex rt-qPCR and Multiplex rt-PCR have been widely used for identifying avian IAVs because they provide better sensitivity and specificity in distinguishing between distinct viral types and subtypes (Abdelwhab et al., 2010). Suwannakarn et al. devised a multiplex rt-qPCR method using TaqMan probes to type and subtype IAVs (Suwannakarn et al., 2008). Wang et al. used multiplex rt-qPCR with SYBR green for the cost-effective subtyping of H5 subtypes (Wang et al., 2009).

#### 5.2.2 DNA microarray

**Figure 8:**
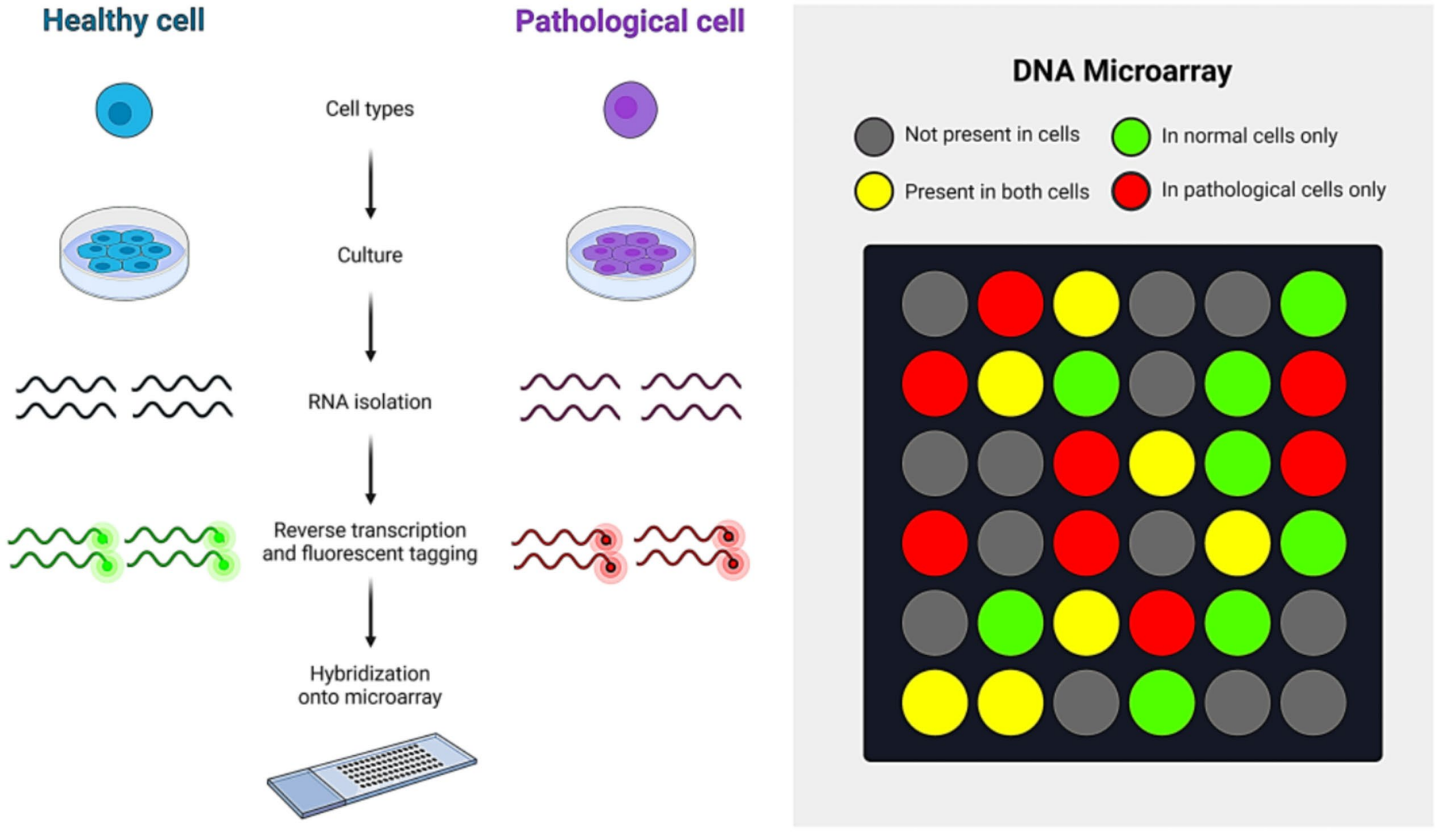
Microarray analysis.

A DNA microarray is a set of oligonucleotides, DNA spots, or probes immobilized on a solid surface. This technique is used for high-throughput, simultaneous wide-range genome screening. To detect IAV, RNA from the sample is first extracted and converted into cDNA. This cDNA is then labeled with a fluorescent dye and hybridized onto a DNA microarray chip. Upon hybridization, the cDNA binds to its complementary sequences on the chip. When the dye is excited, it emits a signal that can be measured using specialized software, providing results on the presence of IAV in the sample (Chugh et al., 2021).The working principle of the DNA microarray technique is shown in Figure 8. Additionally, DNA microarray is crucial for monitoring and medical diagnosis of infectious diseases, including influenza A virus infection. FluChip-55 is a microarray used for IAV typing and subtyping, recognizing 55 influenza virus sequences, including HA, NA, M, and NP genes (Mehlmann et al., 2006). A universal oligonucleotide microarray has been used for almost complete subtyping of IAVs (Ryabinin et al., 2011).

In contrast, MChip is a special microarray for typing and subtyping that can only identify the IAV M gene segment. The MChip employs 15 short oligonucleotides for hybridization. The pattern of fluorescence signal intensity in each array is used to determine the IAV subtype (Sakurai and Shibasaki, 2012). DNA microarray technology has some limitations, including the high cost of equipment and the specialized skills needed to perform the technique. Additionally, it requires a specific quantity of cDNA, which may necessitate the propagation of the virus in cultured cells or embryonated chicken eggs. Despite these challenges, DNA microarray remains a highly effective method for detecting IAV due to its high-throughput capability.

#### 5.2.3 Next-generation sequencing

Sanger sequencing, introduced in 1977, is widely utilized for DNA detection but has limitations like long reaction time, low throughput, and high cost. In contrast, next-generation sequencing (NGS), introduced in the late 1990s, employs pyrophosphate sequencing, a high-throughput non-gel method. High-throughput sequencing provides sensitivity and throughput but is time-consuming, expensive, and primarily used for research rather than primary diagnostics. Overall, NGS presents a more accurate and efficient approach to DNA detection (Fu et al., 2023). NGS has three types which include Roche 454 GS Junior Titanium (based on pyrosequencing) which was the first platform for NGS, Illumina sequencing (based on reversible chain termination), and Ion Torrent PGM (based on proton detection). The time taken by these methods to process a sample from RNA extraction to results varies as Illumina takes 39 h (Rambo-Martin et al., 2020), Roche 454 GS Junior takes 24 h (Liu et al., 2012), and Ion Torrent PGM takes 33 h (King et al., 2020).

Next-generation sequencing technology can provide untargeted deep sequencing, making it an effective technique for detecting viral subtypes in clinical specimens. Currently, viral genomes are sequenced using three methods based on NGS; PCR amplicon sequencing, target enrichment sequencing, and metagenomic sequencing (Lim et al., 2024). Whole-genome sequencing of IAV can reveal the genetic basis of pathogenicity, anti-viral resistant mutations and identify coinfection or quasispecies. Deep sequencing can also pinpoint the exact etiology of an epidemic in clinical specimens containing trace amounts of viral RNA or DNA. However, turnaround time (TAT) must be considered before whole-genome sequencing is used to investigate epidemics (Shao et al., 2017). Despite its potential for generating full-length IAV genome, NGS technology has some limitations, particularly the production of short reads. Aligning these short reads into a contiguous sequence (contig) can be difficult, especially when the genome contains repetitive sequences, often leading to gaps in the assembly. These gaps are problematic, especially when the repeats exceed the read length or there is inadequate coverage. While aligning the assembled genome with a reference genome sequence might help close these gaps, it becomes more challenging in *de novo* assembly, often requiring advanced bioinformatics tools and a skilled workforce (Alkan et al., 2011). Additionally, due to the segmented RNA genome of IAV, achieving complete genome coverage is challenging (Danasekaran, 2024). Third-generation sequencing has been developed named MinION nanopore sequencing. The benefit of MinION nanopore is the generation of longer reads of about 5,000 bases. The sequencing time for MinION nanopore sequencing is 14.5 h. It is cost-effective, and the data can be analyzed easily (Chauhan and Gordon, 2022).

#### 5.2.4 Loop-mediated isothermal amplification

Notomi et al. developed the LAMP technique in 2000, a unique isothermal nucleic acid amplification method that does not require template heat denaturation, a long-term temperature cycle, or expensive equipment (Notomi et al., 2000). For the LAMP assay, three pairs of primers are typically designed to target specific sequences of the IAV genome. These three pairs of primers include two outer primers, two loop-mediated primers, and two inner primers. The reaction mixture is being carried out at a constant temperature usually running at 60 to 65 C in the presence of *Bst* DNA polymerase. The positive reaction mixture produces magnesium pyrophosphate or fluorescence which is visually detected, making it appropriate for on-site detection (CHENG et al., 2011). Imai et al. developed two internal and two external primers for reverse-transcription loop-mediated isothermal amplification (RT-LAMP), a novel nucleic acid testing method (Imai et al., 2006). Zhang et al. developed RT-LAMP technology, which can filter out avian influenza viruses and identify the H5 and H9 subtypes with a detection limit ten times greater than RT-PCR and a detection time of less than 30 min (Zhang et al., 2020). RT-LAMP detection is a potential diagnostic method for IAV detection since it does not require sophisticated, expensive equipment or experienced workers and is ideal for on-site use. However, it has target-related drawbacks, making the development of specialized primers for the target sequence more difficult. Overall, RT-LAMP detection is a potential diagnostic method for IAV identification, although it is limited in its ability to select particular primers for the target sequence (Fu et al., 2023).

#### 5.2.5 Nucleic acid sequence-based amplification

NASBA is an enzyme-based single-step RNA amplification process that employs avian myelocytic disease virus reverse transcriptase (AMV-RT), ribonuclease H, and T7 RNA polymerase (Deiman et al., 2002). The procedure needs two target-sequence-specific primers, one with a 50-extension containing the RNA polymerase’s T7 promoter sequence. The advantages of NASBA technology include direct recombination of RNA target without reverse transcription into cDNA and selective amplification of RNA sequences in the presence of native genomic DNA (Malek et al., 1994). It has been utilized to identify H5N1 subtype avian IAV and discriminate highly pathogenic ones from mildly pathogenic ones (Collins et al., 2002). The simple amplification-based assay (SAMBA) uses a nitrocellulose dipstick to view test results using the NASBA technique.

SAMBA has detected IAV H1NI in 262 patient samples with 95.3% sensitivity and 99.4% specificity. After isothermal amplification using NASBA, a dipstick was used to visualize the signal across an 85-min test duration (Courtney et al., 2021).

A comparative analysis of these diagnostic techniques is made in Table 1.

**Table 1:** A comparative analysis of the detection techniques and their efficacy.

Method	Time of result	LOD	Sensitivity (%)	Specificity (%)	Advantages	Limitations	References
Hemagglutination	20–30 min	10^5^ -10^6^ 50% egg infectious dose (EID50)/ml	95	100	Simple, inexpensive, quick	Variable incubation time, strain-dependent results	Fu et al. (2023)
Immunochromatography	30 min	5.8 × 10^2^–5.8 × 10^3^ pfu/assay	60	>90	Rapid, easy to use, cost-effective	Low sensitivity, low LOD	Keitel et al. (2011)
RT-qPCR	~240 min	100 copies/reaction	80	100	Rapid, highly sensitive, and specific, recommended	Can not differentiate infectious and noninfectious virion, need specialized expertise, expensive	Nakaya et al. (2020) and Ngoc and Lee (2024)
DNA microarray	less than 100 min	1 × 10^2^ copies/μL	100	98.85	Cost-effective, comprehensive detection, reliable	Cross-reactivity, difficult-to-design probe, low LOD	Sultankulova et al. (2014) and Shen et al. (2019)
NGS	2–3 days	n.s	78	80	full genome sequences allow determination of viral subtype and serotype	High cost, coverage challenges	Thorburn et al. (2015) and Seong et al. (2016)
LAMP	40	10 copies/25 μL (0.4 copies/μL)	100	87.5	Low-cost equipment and minimally trained personnel, well suited for resource-limited settings	Non-specific amplification, complex primer design	Ahn et al. (2019)
NASBA	>120	10 copies/reaction	n.s	n.s	Supports limited subtyping	High background noise, high cost	Fu et al. (2023) and Oliveira et al. (2021)

NGS, next generation sequencing; LAMP, Loop-mediated isothermal amplification; NASBA, Nucleic acid sequence-based amplification; n.s., not specified.

#### 5.2.6 Recent advancements in the diagnosis of IAV

Recent advancements in electrochemical tests for detecting IAV have led to substantial improvements in both sensitivity and specificity. The incorporation of functionalized electrodes with antibodies or aptamers has notably enhanced the performance of these tests. For example, the modification of electrodes with Au/magnetic nanoparticles and carbon nanotubes has resulted in a limit of detection (LOD) as low as 8.4 × 10^−12^ M through DNA hybridization (Lee et al., 2018). Similarly, an electrochemical impedance spectroscopy (EIS) sensor, featuring a nanocrystalline boron-doped diamond electrode combined with an anti-M1 protein polyclonal antibody, has achieved a LOD of 5 × 10^−14^ g/mL for detecting the M1 protein of IAV H1N1 in saliva. Moreover, aptamer-based EIS aptasensors targeting inactivated IAV H1N1 and H3N2 strains have reduced LOD to 0.9 pg./μL (Bai et al., 2018). Lateral flow assays (LFAs), which provide rapid results for IAV detection within approximately 12 min, have also seen significant improvements (Rodriguez et al., 2015). For instance, an antibody-conjugated CdSe/CdS/ZnS QD-latex setup has achieved an LOD of 2.5 HAU/mL for IAV H1N1 and 0.63 HAU/mL for IAV H3N2 (Nguyen et al., 2020). Additional improvements using Au- or Pt-based latex nanoparticles have further lowered LODs, increasing sensitivity across various strains of IAV (Matsumura et al., 2018). The use of thermal contrast amplification (TCA), as well as the integration of convective PCR or RT-LAMP, has enhanced the detection capabilities of LFAs, particularly in the amplification of multiple genes (Wang et al., 2016).

Microfluidic-based assays have emerged as highly flexible platforms for detecting IAV, offering the ability to integrate with a range of detection methods. These systems, such as lab-on-a-CD technology, enable fully automated processes including virus lysis and RT-PCR, thus streamlining the detection workflow. Notably, microfluidic chips that combine hemagglutinin and neuraminidase arrays with glycan-coated magnetic beads have enabled the simultaneous detection of up to 12 different IAV strains within 100 min, achieving LOD as low as 8.5 × 10^−2^ PFU/mL (Zhang et al., 2022). Microarray-based assays, utilizing platforms like paper microarrays or magnetic nano ELISA (MagLISA), have also made significant progress. These platforms offer shorter analysis times, reduced reagent use, and high sensitivity. For example, a paper microarray has achieved an LOD of 2.7 × 10^3^ PFU for IAV H1N1 and 2.7 × 10^4^ PFU for IAV H3N2 (Lei et al., 2015). Other innovations, such as the use of multifunctional quantum dots in digital microarrays, have enabled high-throughput detection of multiple IAV strains with minimal sample preparation (Wu et al., 2019).

Other assays for IAV detection, including surface acoustic wave sensors and capacitive immunosensors, have been developed to offer rapid detection time, with some providing results in as little as 30 s (Gorelkin et al., 2015). Additionally, advancements in electrochemiluminescence (ECL) immunosensors and new signal transduction mechanisms, such as neuraminidase glucose release, have further improved the sensitivity and specificity of IAV detection (Zhang et al., 2022). These developments represent promising pathways for the future of diagnostics of IAV. These innovations demonstrate the ongoing progress in diagnostic assays for influenza A virus detection, with continuous improvements in sensitivity, speed, and the ability to detect multiple targets across various platforms.

#### 5.2.7 Role of artificial intelligence in the diagnosis of IAV

The recent development of artificial intelligence (AI) is transforming the diagnosis of IAV by providing faster, more accurate, and scalable solutions. AI-based systems use machine learning and deep learning algorithms to analyze clinical data, medical images, and genetic sequences, enabling early detection and precise diagnosis. Advanced AI models demonstrate high diagnostic accuracy by analyzing pharyngeal images and integrating clinical information, surpassing traditional methods in efficiency and reliability. These approaches facilitate timely interventions, which are essential for controlling viral transmission and improving patient outcomes (Okiyama et al., 2022). Additionally, AI enhances the analysis of genetic data from next-generation sequencing (NGS), allowing the identification of viral mutations and strain variations crucial for epidemiological studies and vaccine development. AI-driven platforms also play a pivotal role in telemedicine, offering remote diagnostic capabilities that reduce the need for in-person consultations, particularly during outbreaks. By streamlining workflows and minimizing reliance on conventional tests like RT-PCR, AI contributes to faster diagnostics and improved accessibility, especially in resource-limited settings (Ozcelik et al., 2024).

## 6 Public health approach toward influenza A virus

### 6.1 Global surveillance and monitoring

Surveillance and monitoring of IAV are essential to global public health strategies aimed at preventing and controlling outbreaks. The WHO, through its Global Influenza Surveillance and Response System (GISRS), is instrumental in this effort. Established in 1952, GISRS coordinates with National Influenza Centers (NICs) and WHO Collaborating Centers around the world to collect and analyze data on influenza. This network is crucial for the timely identification of circulating strains in the community, which informs vaccine development and public health interventions. Early detection and rapid reporting of IAV outbreaks are vital for implementing targeted containment measures, reducing transmission, and easing the strain on healthcare systems (Bresalier, 2023). For instance, the quick identification of the H1N1 strain in 2009 facilitated the rapid development of vaccines and the implementation of effective public health measures, significantly reducing the pandemic’s impact (Roychoudhury et al., 2020). Thus, ongoing monitoring and global data sharing are crucial for preparing for and responding to potential influenza pandemics.

### 6.2 Vaccination

Influenza vaccines are primarily quadrivalent, targeting two strains of IAV and two strains of IBV, with selection based on factors such as age and risk group. Common egg-based vaccines include inactivated influenza vaccines (IIVs) and live attenuated influenza vaccines (LAIVs) formulations like Afluria, Fluarix, Fluzone, and FluMist. High-dose trivalent IIVs, such as Fluzone High-Dose and Fluad, are recommended for adults aged 65 and older. Recombinant vaccines (RIVs), like Flublok, are produced using mammalian cell line cultures to avoid egg-related mutations and enable faster production, making them suitable for egg-allergic individuals. FluMist Quadrivalent, a nasal spray LAIV, induces mucosal immunity but is not recommended for immunocompromised individuals or children on salicylate medications due to the risk of Reye’s syndrome. IIVs are widely used for their cost-effectiveness and systemic immunity, although they may require booster doses. LAIVs mimic natural infections to induce mucosal immunity, while recombinant hemagglutinin vaccines, produced with insect cells and baculovirus, offer safety during pandemics but require higher antigen levels and are less immunogenic (Nuwarda et al., 2021). Universal influenza vaccines aim to address challenges like antigenic drift and shift by providing long-lasting protection against all influenza A and B subtypes. These vaccines target conserved regions, such as HA, NA, M1, M2, or NP, with the HA stalk region showing promise for neutralizing all influenza A subtypes. Since 2020, mRNA vaccines have shown remarkable success in combating COVID-19, saving countless lives and significantly altering the course of the pandemic. These vaccines provide advantages over traditional inactivated virus vaccines, including faster design, cell-free manufacturing, and the ability to induce strong antibody and cellular immune responses (Arevalo et al., 2022; Chen et al., 2018).

Seasonal vaccination is a key public health measure for mitigating the spread and severity of IAV infections. Each year, flu vaccines are formulated to address the most common strains anticipated to be in circulation, effectively reducing the risk of severe illness, hospitalization, and mortality (Lee et al., 2021). Nevertheless, the high mutation rate of the IAV genome, driven by antigenic drift and shift, presents ongoing challenges for maintaining vaccine effectiveness. These genetic variations can result in discrepancies between the vaccine strains and the circulating viruses, which may reduce the vaccine’s protective efficacy (Ortiz de Lejarazu-Leonardo et al., 2021). To address these issues, research is increasingly directed toward developing universal influenza vaccines that provide broader and more durable protection against a range of influenza strains, including those that may emerge in future pandemics. These vaccines focus on conserved viral components, such as the hemagglutinin stem, which experiences fewer mutations and thus holds promise for overcoming the challenges posed by the virus’s genetic variability (Wei et al., 2020). Continued global surveillance and advancements in vaccine technology are crucial for refining vaccination strategies and ensuring effective protection against IAV.

### 6.3 Antiviral therapies

Antiviral drugs are important for treating active IAV infection, as they help to reduce the severity and duration of illness when administered early. However, the rise of antiviral-resistant IAV strains is an increasing concern. M2 ion channel targeting drugs (amantadine and rimantadine) were once highly effective against IAV, with efficacy rates reaching 90%. However, resistance rose significantly after 2000, and by 2013, 45% of circulating IAVs were resistant due to the S31N mutation in the M2 protein. As a result, these drugs are no longer recommended (Chakraborty and Chauhan, 2024). Neuraminidase inhibitors (NAIs), now the primary treatment for IAVs, reduce illness duration by 30–44% if administered within 24-36 h of symptom onset and lower complications like pneumonia and otitis media. Oseltamivir resistance was rare until 2007–2008 when H1N1 strains emerged with human-to-human transmissibility, reaching resistance rates over 90% in regions like the USA, Canada, and the UK by 2008–2009. H1N1pdm09 strain was initially susceptible to oseltamivir, but resistance later arose in immunocompromised individuals due to mutations like H275Y in H1N1 and R292K in H3N2, reducing efficacy against oseltamivir and zanamivir. Baloxavir marboxil use has led to the PA/I38X mutation in 9.7% of patients within 3–9 days, resulting in prolonged symptoms and higher viral loads. Combination therapies, such as amantadine, oseltamivir, and ribavirin, show synergistic effects *in vitro* and animal models. A clinical trial involving naproxen, oseltamivir, and clarithromycin for influenza A(H3N2) demonstrated reduced hospital stays and improved survival rates, supporting naproxen’s potential in antiviral strategies (Raza and Ashraf, 2024). Moreover, there is an urgent need to develop new antiviral drugs with different mechanisms of action to address resistance and expand treatment options. Research is actively exploring combination therapies and broad-spectrum antivirals to strengthen the therapeutic arsenal against resistant strains of IAV.

### 6.4 Nonpharmaceutical interventions

Non-pharmaceutical interventions (NPIs) are vital in managing the spread of IAV, especially when vaccination and antiviral treatments are not readily available. Public health campaigns that emphasize hygiene practices such as regular handwashing, mask-wearing, and proper respiratory etiquette are effective in limiting transmission. Research has shown that these measures can significantly reduce influenza incidence by minimizing the dissemination of respiratory droplets and viral particles (Le Sage et al., 2023). Additionally, social distancing strategies, including quarantine, isolation, and maintaining physical distance, are crucial for controlling outbreaks. Evidence from the 2009 H1N1 pandemic indicates that such measures played a key role in curbing the virus’s spread and alleviating pressure on healthcare systems (Fong et al., 2020). Applying these NPIs based on timely and accurate surveillance data ensures they are tailored to the current epidemiological situation, thereby enhancing their effectiveness and reducing the overall impact of IAV on public health.

### 6.5 Pandemic preparedness and response

Effective management of influenza A outbreaks relies on robust pandemic preparedness plans, which include strategic resource allocation, strengthening healthcare systems, and implementing comprehensive public communication strategies. A well-developed emergency response plan enables prompt and coordinated actions, such as distributing vaccines and antivirals, improving healthcare infrastructure, and executing public health measures (Haw et al., 2022). Strengthening the healthcare system requires expanding surge capacity, maintaining sufficient stockpiles of medical supplies, and training healthcare workers to manage increased patient volumes during outbreaks. Additionally, effective public communication is crucial for disseminating accurate information about the virus and preventive measures, which helps foster public compliance and mitigate misinformation. Interdisciplinary collaboration among governments, healthcare systems, and the public is essential for a unified response. Such collaboration integrates expertise across various sectors, optimizes resource use, and ensures that response strategies are adapted to the changing dynamics of the pandemic (Bendowska and Baum, 2023). This coordinated approach is key to enhancing pandemic resilience and effectively controlling IAV.

### 6.6 Public education and awareness

Effective risk communication is crucial for managing IAV outbreaks and ensuring public adherence to preventive measures. Employing a range of communication channels such as social media, traditional media, and community outreach is vital for disseminating clear, concise, and actionable information about the virus and preventive practices (de Sa et al., 2009). Engaging trusted sources, including public health authorities and healthcare professionals, is important for establishing credibility and building public trust. At the same time, addressing misinformation is essential to avoid confusion and reduce vaccine hesitancy. Recent studies emphasize the importance of proactive monitoring of misinformation and implementing targeted strategies to counter false claims and provide accurate information (Shahbazi and Bunker, 2024). Efforts to manage misinformation should concentrate on correcting inaccuracies through reliable sources and ensuring that accurate information is readily available, thereby supporting public health initiatives and improving the effectiveness of response efforts.

### 6.7 Research and development

Ongoing research into IAV is crucial for understanding the virus’s evolution, transmission dynamics, and developing effective vaccines and treatments. Continuous surveillance and genetic analysis are vital for monitoring changes in the virus, which can affect vaccine efficacy and treatment strategies. Research efforts are directed toward identifying new viral strains, examining their transmission patterns, and enhancing vaccine formulations to address issues like antigenic drift and shift (Lim et al., 2024; Shao et al., 2017). Furthermore, the one health approach underscores the interconnectedness of human, animal, and environmental health in managing zoonotic diseases such as IAV infections. This approach integrates data from health science, veterinary science, ecology, and environmental science to better understand how changes in animal reservoirs, environmental conditions, and human activities contribute to the emergence and spread of IAVs (Danasekaran, 2024). By incorporating these diverse perspectives, the one health approach improves our ability to predict and mitigate the impact of IAV outbreaks, leading to a more comprehensive and proactive public health response.

### 6.8 Global and national policies

National and international policies are vital in shaping public health responses to IAV outbreaks, guiding essential measures such as travel restrictions, trade regulations, and vaccination mandates. Frameworks provided by international bodies like the WHO offer guidelines for managing cross-border health threats and facilitate coordinated responses to global pandemics. National policies adapt these guidelines to local contexts, implementing travel restrictions to curb virus spread, regulating trade to ensure medical supply safety, and mandating vaccinations to achieve high coverage and herd immunity (Leask et al., 2021). Addressing equity in health access is equally important, as disparities in vaccine and treatment availability can worsen the impact of IAV in low-resource settings. Ensuring equitable distribution involves tackling logistical challenges, financial barriers, and infrastructure limitations that disproportionately affect vulnerable populations. Recent research highlights the necessity for global collaboration and funding mechanisms to support equitable access, aiming to mitigate health inequities and strengthen overall pandemic preparedness (Lal et al., 2022).

## 7 Conclusion

The isolation and identification of influenza A viruses are vital for understanding their epidemiology, pathogenesis, and developing effective countermeasures. This article has outlined various approaches for the isolation and characterization of IAV strains from diverse sources, ranging from traditional cell culture-based methods to advanced techniques like RT-qPCR, NGS, and nucleic acid-based assay. Each of these methods contributes to an increasingly sophisticated toolkit for diagnosing and detecting IAV. Recent advancements in point-of-care detection technologies have further revolutionized IAV diagnosis, offering cost-effective solutions. Advances in diagnostic techniques, particularly NGS and nucleic acid sequencing-based methods, have significantly enhanced the sensitivity and specificity of IAV strain identification. These technological improvements have strengthened our preparedness for seasonal outbreaks and enhanced our capacity to respond swiftly to potential pandemics. As our understanding of IAV deepens, ongoing research continues to explore the complex interactions between viral components and host responses, shedding light on factors that contribute to virulence and immune evasion. To address the ongoing threat posed by continually mutating IAV strains, a holistic and multidisciplinary approach is necessary. This approach should extend beyond diagnostic labs to engage the broader community in effective infection control. By integrating virology, epidemiology, immunology, and computational biology, we can identify novel therapeutic targets, enhance vaccine development, and more effectively engage the community, ultimately improving our ability to combat future outbreaks.
